# Road Recognition Based on Vehicle Vibration Signal and Comfortable Speed Strategy Formulation Using ISA Algorithm

**DOI:** 10.3390/s22176682

**Published:** 2022-09-03

**Authors:** Xiulai Wang, Zhun Cheng, Ningling Ma

**Affiliations:** 1 Medical Information Center, Jinling Hospital, Nanjing 210002, China; 2 Department of Vehicle Engineering, Nanjing Forestry University, Nanjing 210037, China

**Keywords:** vehicle vibration, signal processing, comfortable, road roughness, road recognition, ISA algorithm

## Abstract

When a vehicle is being driven, it is excited by the road roughness and generates its own vibration. In order to improve the vehicle’s riding comfort and the physical–mental health of passengers in the vehicle, this paper proposes a formulation method for a comfortable speed strategy and the technical route of its application. According to international standard ISO 2631-1, the relationship between the weighted root-mean-square acceleration value and comfortable vehicle speed is analyzed. The simulation test platform of the road roughness signal and vehicle vibration signal is built by using the filtering white noise method and the second Lagrange equation through Matlab/Simulink. Combined with the simulation platform, this paper extracts seven characteristics with statistical properties from the time-domain signal and obtains 500 sample data. Random forest (RF), extreme learning machine (ELM), and radial basis function neural network (RBF-NN) are applied to identify roads. Two comfortable speed strategy formulation methods based on the improved simulated annealing (ISA) algorithm are proposed and compared according to the solution effect of each grade of comfortable speed. The results show that the simulated signals of each grade road roughness are accurate. Road recognition can be effectively carried out using the statistical characteristics of vehicle vibration acceleration signals. ELM has high recognition accuracy and fast execution speed. The ISA-II algorithm has a low solution error of comfortable speed and a low computation time. The comfortable speed of the research vehicle on different road grades showed a great difference.

## 1. Introduction

Vehicles are among the most widely used tools for transportation. Even if the main role of some vehicles is not transportation (agricultural vehicles [1,2], engineering vehicles [3], etc.), they always drive on various types of roads (mainly including hard roads and soft soil roads [4]). As a source of external excitation of the vehicle, the uneven height of the driving road will cause vehicle vibration [5,6]. Under the action of this incentive, the reliability of each vehicle’s subsystem, the riding comfort, and even the health condition of the passengers will be affected to a certain extent. Therefore, it is of great significance to explore the relationship between the subjective feelings of passengers in vehicles and the vehicle behavior.

The vibration felt by the passengers in the vehicle is caused by the road roughness excitation and transmitted through each subsystem of the vehicle. Therefore, the research on vehicle vibration characteristics mainly has two directions, namely, road roughness and vehicle subsystems related to ride comfort. The research on road roughness is mainly oriented toward the measurement, simulation, and identification of road roughness. Cheng and Lu [7] used a noncontact road roughness measuring instrument to obtain the road roughness signal. This study explored the method of compressively collecting the road roughness signal. Lu et al. [8] designed a surface roughness instrument (the test accuracy was within the accuracy range of ±2 mm) and carried out the corresponding surface roughness measurement test. Some scholars adopted the vehicle dynamic response to obtain (measure or identify) the road roughness signal. For example, Gonzalez et al. [9] established a half-vehicle vibration model without a driver-seat system and measured the axle and body accelerations using computer simulation technology. This study used a transform function to correlate vehicle vibration acceleration with road roughness. It was verified that this road recognition method had accurate estimation accuracy. Yousefzadeh et al. [10] proposed an artificial neural network to estimate road roughness. This study built a vibration simulation model of an off-road vehicle based on ADAMS software to obtain the training data required for the neural network. The vehicle vibration signals used in Fauriat et al. [11] were mainly body acceleration and wheel acceleration. This study developed a road estimation algorithm based on Kalman filtering theory. Liu et al. [12] estimated road roughness information using the augmented Kalman filtering algorithm and used the international roughness index as the basis for road classification. This study used a half-vehicle vibration model with four degrees of freedom and ADAMS software. The research of Li et al. [13] was based on a half-vehicle vibration model with four degrees of freedom. This study analyzed the accuracy of road roughness recognition using orthogonal tests and NARX neural networks. It can be seen that these methods were based on vehicle simulation data or actual vibration test data, combined with neural networks, Kalman filtering theory, and other methods, to estimate road conditions.

The extant research on vehicle subsystems related to smoothness focused on the improvement and evaluation of vehicle ride comfort. For example, the research of Zhao and Wu [14] used ADAMS software and the nondominated sorting genetic algorithm II. This study took the maximum vertical acceleration at the seat as the improvement target of vehicle smoothness. Sharma et al. [15] used the random search technique to optimize ride comfort with the weighted vertical and lateral RMSAR (root-mean-square acceleration response) of the sprung mass center as the objective function. Kaldas et al. [16] proposed a new comprehensive objective function and combined with the application of the damper top mounts to improve the ride comfort and harshness of the vehicle. The new comprehensive objective function used in this study mainly involved the physical quantities related to the vertical acceleration. The evaluation index included parameters of relative importance between variables. Chen et al. [17] pointed out that most of the current research mainly used the weighted root-mean-square acceleration value to analyze riding comfort. This study also used the weighted root-mean-square acceleration value as the primary objective function for the smoothness optimization of heavy vehicles. The research of Chen et al. [18] used the weighted root-mean-square acceleration value as an index for evaluating vehicle riding comfort, as well as for comparing simulation models with test results. This study selected wheel dynamic load and the weighted root-mean-square acceleration as optimization goals in the process of improving vehicle riding comfort. These studies took the vehicle ride comfort evaluation index (such as weighted root-mean-square acceleration value) as the objective function, and then combined it with vehicle simulation technology and an optimization algorithm to optimize the design of vehicle subsystem related to smoothness. In addition, some studies focused on the riding comfort of the passengers in the car. For example, Li et al. [19] analyzed and improved the riding comfort of an ambulance considering patient health and driver comfort. In this research, the multi-freedom vibration model of the vehicle and the multi-objective optimization method were adopted. Cieslak et al. [20] combined neural network technology, human body measurement data, and acceleration measurement data to explore the riding comfort of occupants in the car.

On the basis of the above discussion, the method of using specific instruments to measure road roughness has disadvantages in terms of cost (the price of instrument) and time consumption (the test speed is relatively slow), While the method of using the vehicle system dynamic response to estimate road roughness has advantages in terms of cost and time consumption. At present, there are abundant research reports on this method in the identification and estimation of road roughness (time-domain and frequency-domain) signal. However, the application of this method in road classification is still insufficient. According to [9,10,11,12], there are many studies on half-vehicle vibration models with the four degrees of freedom. The model with four degrees of freedom mostly adopts the vehicle body vibration acceleration or wheel vibration acceleration. This leads to the difficulty of sensor installation and measurement in practical applications. This road classification method usually needs to measure a variety of physical quantity signals during use. Moreover, road roughness usually needs to be estimated before road grade classification. However, the road roughness signal is random, which has statistical significance. Therefore, it has practical value if it can be combined with vehicle vibration acceleration signals for direct road classification. Comparative studies of various types of identification methods are also needed. In addition, the current research on vehicle ride comfort focuses on the improvement design of vehicle-related subsystems. However, when the vehicle manufacturing is completed, its vibration characteristics and ride performance have basically been determined (in general). This leads to the fact that there are always bad roads that make the vehicle vibrate greatly when driving, thus affecting the comfort and physical–mental health of passengers in the vehicle. Therefore, vehicle behavior analysis is carried out for completed vehicles (i.e., the vehicle speed is calculated according to the current road type and comfort evaluation indices) to improve vehicle performance. There is a nonlinear complex relationship among road roughness, subjective feelings of passengers in the vehicle, vehicle vibration characteristics, and vehicle speed. This makes it difficult to solve the engineering problem.

In order to solve the above problems and improve ride comfort, this paper studies and proposes a comfortable speed strategy and the technical route of its application. The research work of this paper is mainly divided into three parts: (1) building a simulation test platform for the road roughness signal and vehicle vibration signal to provide sample data for research and verification; (2) combined with the statistical characteristics of the signal and three methods (random forest (RF), extreme learning machine (ELM), and radial basis function neural network (RBF-NN)), the road recognition system is established and compared; (3) using the improved simulated annealing (ISA) algorithm, two comfortable speed strategy formulation methods are proposed and compared. The research work of this paper is expected to provide a valuable reference and direct help for road recognition, vehicle vibration characteristic assessment, vehicle suspension system design, speed recommendation, and speed control auxiliary decision information (for drivers or autonomous driving systems), as well as other aspects.

## 2. Materials and Methods

### 2.1. Definition of Comfortable Speed and Technical Route of Strategy Application

There is a strong correlation between the vehicle driving smoothness and the riding comfort of the passengers. When the vibration of the vehicle during driving is too large (i.e., exceeding a certain threshold), the passengers will obviously feel uncomfortable. This also has a significant impact on the health of passengers in the vehicle. In particular, it is more necessary to pay attention to the vehicle ride comfort for an ambulance or a vehicle transporting an injured person.

In general, when the vehicle manufacturing is completed, its vibration characteristics have basically been determined (since the vibration characteristics parameters of the vehicle are basically unchanged). A number of studies (e.g., Jin [21], Wang et al. [22], Gao and Zhang [23], Wang and Easa [24], and Gedik et al. [25]) showed that vehicle speed is positively correlated with vibration acceleration, and there is a nonlinear relationship between them. International standard ISO 2631-1 (namely *mechanical vibration and shock*—*evaluation of human exposure to whole*-*body vibration*) is one of the main standards used in relevant international studies (this standard has been widely used; e.g., Eger et al. [26], Delcor et al. [27], De la Hoz-Torres et al. [28], and Zhao et al. [29]). ISO 2631-1 clearly gives the basic evaluation method of passengers’ subjective feelings (i.e., using weighted root-mean-square acceleration value as the evaluation index) and the relationship between weighted root-mean-square acceleration value and people’s subjective feelings (Table 1).

The calculation formula of weighted root-mean-square acceleration value is as follows (referring to ISO 2631-1):

(1) $$a_{w}={\left[\frac{1}{T}\int_{0}^{T}a_{w}^{2}(t)dt\right]}^{0.5},$$

where $a_{w}$ is weighted root-mean-square acceleration value, T is the analysis time of vibration, and $a_{w}^{}(t)$ is the time history of the weighted acceleration signal, which is obtained from the time history of the acceleration signal a(t) through the filtering network of the corresponding frequency weighted function w(f).

The calculation formula of the frequency-weighted function is as follows [30,31]:

(2) $$w(f)=\{\begin{array}{ll} 0.5\; & \left(0.5<f<2\right)\\ f/4 & \left(2<f<4\right)\\ 1 & \left(4<f<12.5\right)\\ 12.5/f & \left(12.5<f<80\right) \end{array},$$

where f is the frequency (Hz).

In summary, for the same road grade (see Section 2.2 for details regarding the road grade), the vibration acceleration of the same vehicle is mainly determined by the speed. In this paper, the lower limit of the weighted root-mean-square acceleration value (0.315, 0.5, 0.8, 1.25, 2 m/s^2^) corresponding to each comfort level is defined as the comfortable speed of each level, and a total of five levels of comfortable speed were set to $u_{a1}$–$u_{a5}$, corresponding to the vehicle speed when the weighted root-mean-square acceleration values were 0.315, 0.5, 0.8, 1.25, and 2 m/s^2^, respectively. The specific significance of $u_{a1}$–$u_{a5}$ are as follows: (1) when the vehicle speed is lower than $u_{a1}$, the passengers in the vehicle do not feel uncomfortable; (2) when the speed is at $u_{a1}$–$u_{a2}$, the passengers in the vehicle feel some discomfort; (3) when the speed is at $u_{a2}$–$u_{a3}$, the passengers in the vehicle feel quite uncomfortable; (4) when the speed is at $u_{a3}$–$u_{a4}$, the passengers in the vehicle feel uncomfortable; (5) when the speed is at $u_{a4}$–$u_{a5}$, the passengers in the vehicle feel very uncomfortable; (6) when the speed is higher than $u_{a5}$, the passengers in the vehicle feel extremely uncomfortable. Therefore, $u_{a1}$–$u_{a5}$ is the judgement basis for the comfort and health status of the passengers which feel ‘some discomfort’, ‘quite uncomfortable’, ‘uncomfortable’, ‘very uncomfortable’, and ‘extremely uncomfortable’.

Accordingly, this paper proposes a comfortable speed strategy to provide speed recommendations for drivers or automatic driving system vehicles in the current state (speed state and road roughness state), as well as to provide auxiliary decisions for speed control.

**Figure 1:**
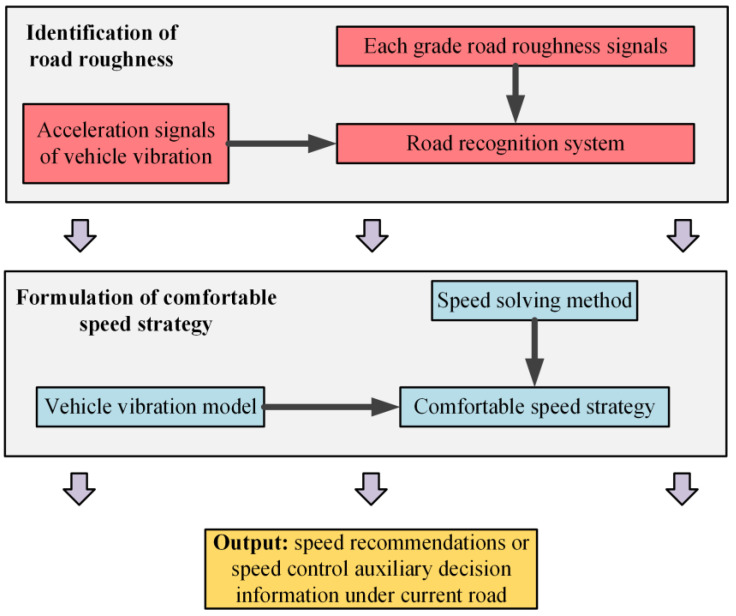
The research technical route of the comfortable speed strategy proposed in this paper.

Specifically, the research technical route of the comfortable speed strategy proposed in this paper is shown in Figure 1.

### 2.2. Simulation of Road Roughness Signal

The road roughness signal has a certain degree of randomness. Therefore, the road power spectral density is mainly used to describe its statistical characteristics. According to international standards ISO 8608 (namely, *mechanical vibration*—*road surface profiles*—*reporting of measured data*), road roughness is usually divided into several levels (Table 2). ISO 8608 suggests using the following fitting expression to calculate the road power spectral density:

(3) $$G_{q}(n)=G_{q}(n_{0}){\left(\frac{n}{n_{0}}\right)}^{-w},$$

where n is spatial frequency ($m^{-1}$), $n_{0}$ is the reference spatial frequency (0.1 generally), $G_{q}(n_{0})$ is the power spectral density at the reference spatial frequency $n_{0}$ ($m^{3}$), also known as the road roughness coefficient, and w is the frequency index (2 generally).

The key to studying vehicle vibration characteristics and road roughness is to simulate different grades of road roughness signals and establish a road roughness model [32,33]. At present, the filtering white noise method, harmonic superposition method, inverse Fourier-transform method, and time series modeling method are all examples of methods used to simulate road roughness signals and establish road models in the time domain. Among them, the filtering white noise method converts the suitable white noise signal into road roughness signals in the time domain, which was used by most researchers to establish a road time-domain model (e.g., Shi et al. [34], Chen et al. [35], and Yin et al. [36]).

Therefore, this paper uses a filtering white noise method to simulate the roughness signals of each road grade. Let the vehicle speed be $u_{a}$; then, the conversion formula of time frequency f and spatial frequency n is as follows:

(4) $$f=u_{a}n.$$

Since the power spectral density refers to the power in the unit frequency range, the spatial frequency spectral density and the time frequency spectral density can be expressed as follows:

(5) $$G_{q}\left(n\right)=\begin{array}{cc} \underset{\Delta n\to 0}{\lim} & \frac{\sigma_{q∼\Delta n}^{2}}{\Delta n} \end{array},$$

(6) $$G_{q}\left(f\right)=\begin{array}{cc} \underset{\Delta f\to 0}{\lim} & \frac{\sigma_{q∼\Delta n}^{2}}{\Delta f} \end{array},$$

where $\sigma_{q∼\Delta n}^{2}$ is the power contained in the road power spectral density in frequency range Δn.

On the basis of the above formulas, it can be deduced that

(7) $$G_{q}\left(f\right)=\frac{1}{u_{a}}G_{q}\left(n\right).$$

Combined with angle frequency ω=2πf, it can be obtained that

(8) $$G_{q}\left(\omega \right)=4\pi^{2}G_{q}\left(n_{0}\right)n_{0}^{2}\frac{u_{a}}{\omega^{2}}.$$

If the cutoff frequency is $\omega_{0}$, then

(9) $$G_{q}\left(\omega \right)=4\pi^{2}G_{q}\left(n_{0}\right)n_{0}^{2}\frac{u_{a}}{\omega_{0}^{2}+\omega^{2}}.$$

Assuming that the above equation is the response of the first-order linear system under white noise excitation, the frequency response function can be set as follows:

(10) $$H\left(j\omega \right)=\frac{a}{b+j\omega },$$

where a and b are unknown coefficients to be solved.

In general, they are combined in the following formula:

(11) $$G_{q}\left(\omega \right)={\left|H\left(j\omega \right)\right|}^{2}S_{\omega },$$

where $S_{\omega }$ is the white noise signal.

Finally, it can be derived that $a=2\pi n_{0}\sqrt{G_{q}\left(n_{0}\right)u_{a}}$, $b=\omega_{0}$. The differential equation of the system can be derived from the frequency response function of the system.

(12) $$2\pi n_{0}\sqrt{G_{q}\left(n_{0}\right)u_{a}}\cdot \omega \left(t\right)=\omega_{0}q\left(t\right)+\dot{q}\left(t\right),$$

where ω(t) is time-domain signal of Gaussian white noise, q(t) is the time-domain signal of the road roughness grade, and $\omega_{0}=2\pi n_{00}u_{a}$, $n_{00}$ is the road cutoff spatial frequency (0.01 m^−1^) [36].

**Figure 2:**
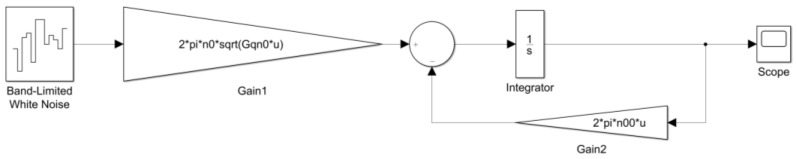
Road roughness signal simulation model built using Matlab/Simulink.

In this paper, Matlab/Simulink was used to build a simulation model of the A–H grade road roughness signals based on the filtering white noise method, as shown in Figure 2.

Since the road roughness signal is the external excitation that causes vehicle vibration, the simulation effect of different grade road roughness signals directly affects the accuracy of this study. Therefore, this study verified the simulated road roughness signal according to the road classification principle proposed in the international standard ISO 8608. The specific steps of verification were as follows: (1) the simulated road roughness signal in the time domain was converted to the road spatial power spectral density; (2) the road spatial power spectral density was expressed as a double-logarithmic coordinate; (3) it was compared and verified whether the power spectrum of each road grade was within the upper and lower limits of the standard road. If the simulated road power spectrum was within the upper and lower limits of the standard road, the simulated road roughness signal met the requirements.

### 2.3. Construction of Vehicle Vibration Signal Simulation Test Platform

**Figure 3:**
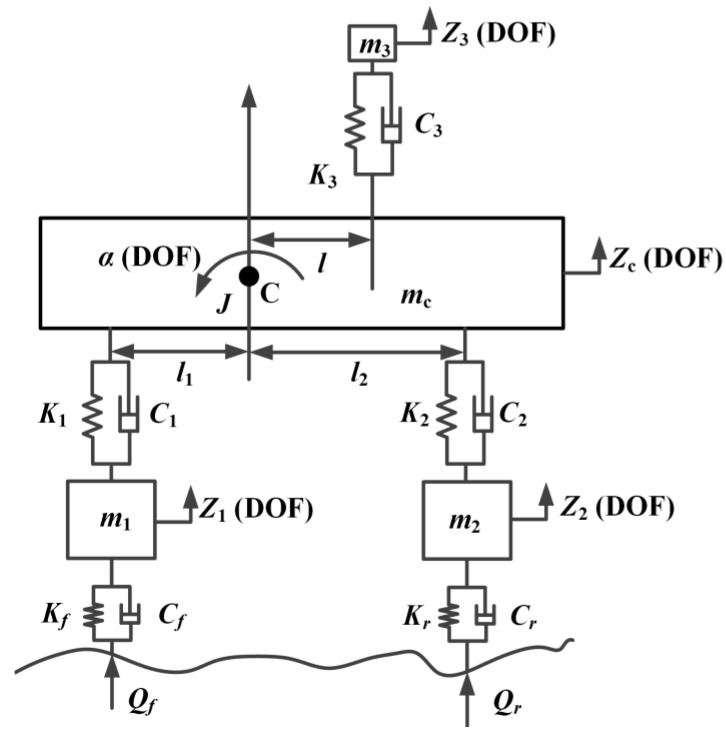
The schematic diagram of the half-vehicle vibration model with five degrees of freedom.

In this paper, the second Lagrange equation was used to establish the vehicle vibration model and simulate the vehicle vibration signal. In general, the vehicle can be considered symmetrical about its longitudinal axis in the research process. This paper assumed that the wheels on both sides of the vehicle experienced the same road roughness during driving. Therefore, the whole vehicle model could be simplified as a half-vehicle model. The half-vehicle model had five degrees of freedom: vertical vibration freedom at the driver’s seat, vertical vibration freedom at the centroid of the body (on behalf of the sprung mass), pitching angle vibration freedom at the centroid of the body, vertical vibration freedom of the front wheel (on behalf of one of the unsprung masses) and vertical vibration freedom of the rear wheel (on behalf of one of the unsprung masses). In addition, due to the wide use of biaxial vehicles, this paper took the biaxial vehicle as the representative vehicle. A schematic diagram of the half-vehicle vibration model with five degrees of freedom is shown in Figure 3.

The definitions of variables and symbols in Figure 3 are as follows: C is the centroid position of the body; *Z*_1_, *Z*_2_, *Z*_3_, *Z_c_*, and *α* are the vertical freedom of front wheel system (translation), vertical freedom of rear wheel system (translation), vertical freedom of driver-seat system (translation), vertical freedom of sprung mass (translation), and body pitching angle freedom (rotation), respectively; *m*_1_–*m_c_* are the mass of the front wheel system, rear wheel system, driver-seat system, and sprung portion, respectively; *J* is the moment of inertia of the sprung portion; *l*_1_ is the distance between the centroid and front axis; *l*_2_ is the distance between the centroid and rear axis; *l* is the distance between the centroid and driver-seat system; [*C*_1_, *C*_2_, *C*_3_, *C_f_*, *C_r_*] and [*K*_1_, *K*_2_, *K*_3_, *K_f_*, *K_r_*] are the stiffness and damping of each subsystem (namely, front suspension, rear suspension, seat system, front wheel, and rear wheel); *Q_f_* and *Q_r_* are the road roughness excitation signals received by the front and rear wheels, respectively.

The expression of the second Lagrange equation is as follows:

(13) $$\frac{d}{dt}(\frac{\partial L}{\partial {\dot{q}}_{i}})-\frac{\partial L}{\partial q_{i}}+\frac{\partial U}{\partial {\dot{q}}_{i}}=Q_{i},$$

where *q* is the generalized coordinate, L=T−V is the difference between kinetic energy T and potential energy V, U is the dissipated energy, usually referring to the energy lost by the damping element, and *Q_i_* is the generalized force.

According to the second Lagrange equation, the vibration differential equation of the half-vehicle vibration with five degrees of freedom is as follows:

(14) $$M\ddot{x}+C\dot{x}+Kx=K_{Q}Q+C_{Q}\dot{Q}.$$

(15) $$M=\left[\begin{array}{lllll} m_{c} & 0 & 0 & 0 & 0\\ 0 & J & 0 & 0 & 0\\ 0 & 0 & m_{1} & 0 & 0\\ 0 & 0 & 0 & m_{2} & 0\\ 0 & 0 & 0 & 0 & m_{3} \end{array}\right].$$

(16) $$K=\left[\begin{array}{lllll} k_{1}+k_{2}+k_{3} & -l_{1}k_{1}+k_{2}l_{2}+k_{3}l & -k_{1} & -k_{2} & -k_{3}\\ -l_{1}k_{1}+k_{2}l_{2}+k_{3}l & k_{1}l_{1}^{2}+k_{2}l_{2}^{2}+k_{3}l^{2} & k_{1}l_{1} & -k_{2}l_{2} & -k_{3}l\\ -k_{1} & k_{1}l_{1} & k_{1}+k_{f} & 0 & 0\\ -k_{2} & -k_{2}l_{2} & 0 & k_{2}+k_{r} & 0\\ -k_{3} & -k_{3}l & 0 & 0 & k_{3} \end{array}\right].$$

(17) $$C=\left[\begin{array}{lllll} c_{1}+c_{2}+c_{3} & -l_{1}c_{1}+c_{2}l_{2}+c_{3}l & -c_{1} & -c_{2} & -c_{3}\\ -l_{1}c_{1}+c_{2}l_{2}+c_{3}l & c_{1}l_{1}^{2}+c_{2}l_{2}^{2}+c_{3}l^{2} & c_{1}l_{1} & -c_{2}l_{2} & -c_{3}l\\ -c_{1} & c_{1}l_{1} & c_{1}+c_{f} & 0 & 0\\ -c_{2} & -c_{2}l_{2} & 0 & c_{2}+c_{r} & 0\\ -c_{3} & -c_{3}l & 0 & 0 & c_{3} \end{array}\right].$$

(18) $$K_{Q}=\left[\begin{array}{ll} 0 & 0\\ 0 & 0\\ -k_{f} & 0\\ 0 & -k_{r}\\ 0 & 0 \end{array}\right].$$

(19) $$C_{Q}=\left[\begin{array}{ll} 0 & 0\\ 0 & 0\\ -c_{f} & 0\\ 0 & -c_{r}\\ 0 & 0 \end{array}\right].$$

(20) $$x={\left[\begin{array}{lllll} Z_{c} & \alpha & Z_{1} & Z_{2} & Z_{3} \end{array}\right]}^{T}.$$

(21) $$Q={\left[\begin{array}{cc} Q_{f} & Q_{r} \end{array}\right]}^{T}.$$

**Figure 4:**
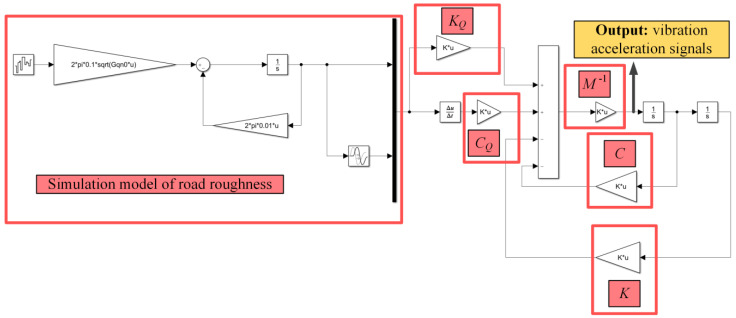
The vehicle vibration signal simulation test platform.

The vehicle vibration signal simulation test platform with five degrees of freedom based on Matlab/Simulink is shown in Figure 4.

### 2.4. Road Recognition Method Based on Time-Domain Signal of Seat Vibration Acceleration

**Figure 5:**
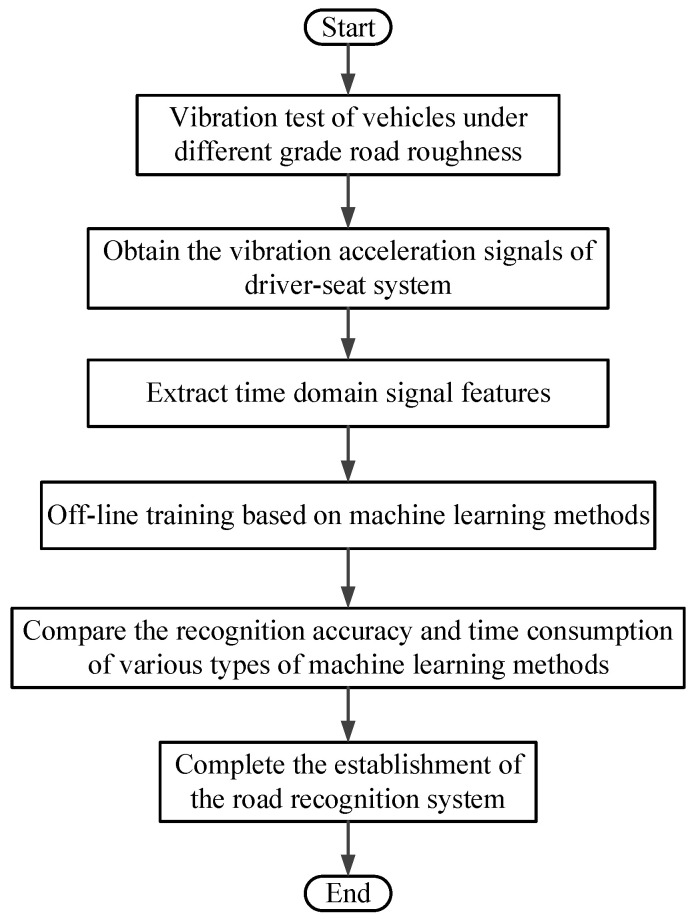
The flowchart of the road recognition system established using the proposed method.

In general, the road roughness signals need to be fully measured or estimated, and then its grade can be determined through the power spectral density. The international roughness index (IRI) is also an indirect method for identifying road grade [12]. This method refers to the cumulative absolute value of the suspension dynamic deflection per unit mileage at a driving speed of 80 km/h. In this paper, a road recognition method based on the time-domain signal of seat vibration acceleration is proposed. It is relatively easy to measure the time-domain signal of driver-seat system vibration, and only one vibration acceleration sensor is used in this process. This method establishes the road recognition system through offline learning of training samples with the help of a machine learning method. This facilitates further improvement of the effect and speed of road recognition in practical use. The flowchart of the road recognition system established using this method is shown in Figure 5.

Specifically, this research adopted RF [37], ELM [38], and RBF-NN [1] to construct the road recognition system.

**Figure 6:**
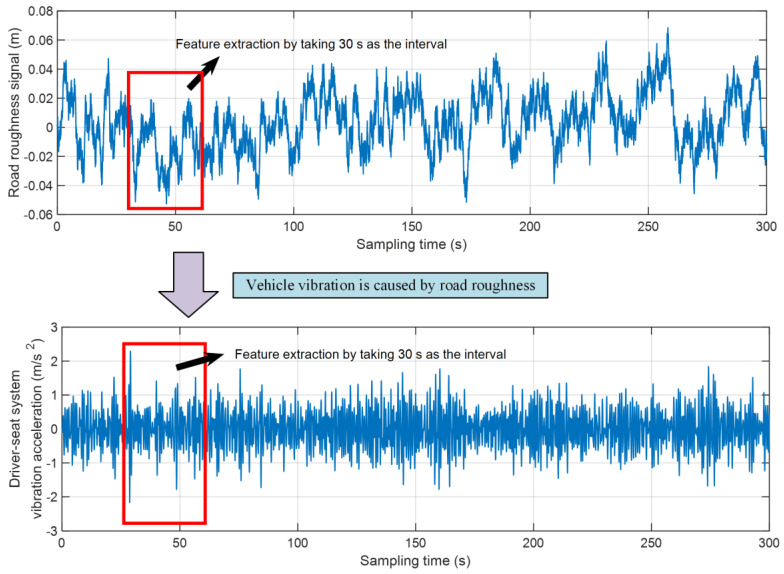
The schematic diagram of feature extraction.

Road roughness excitation will cause vehicle vibration. This research constructed the road recognition system by combining the vibration signal of the driver-seat system with three methods (RF, ELM, and RBF-NN). This paper studied the feature extraction of vehicle vibration signals under different road grades (taking A–E grade roads as examples). The vehicle vibration signals under different grade roads were divided using a 30 s interval of sampling time. A schematic diagram of the division process is shown in Figure 6.

This research obtained 3000 s of signals for each grade road, i.e., 100 sample data for each grade road. This paper took A–E grade roads as examples. Thus, a total of 500 sample data were obtained. Among them, 400 sample data were used as the training set, and the remaining 100 sample data were used as the test set.

According to the statistical characteristics of the road roughness signal, vehicle vibration is caused by road roughness. Therefore, the vehicle vibration signal also has statistical characteristics, and the number of vibration time-domain signals within 30 s (i.e., one sample datum) is large. Combined with the above characteristics of the measured signal, this study took the average value of the absolute value, the standard deviation of the absolute value, the maximum deviation of absolute value from the average value of the absolute value, the extreme difference of the absolute value, the weighted root-mean-square acceleration value, the average value, and the standard deviation of each sample datum as the seven characteristics ($f_{e1}$–$f_{e7}$, respectively) of each sample datum (the vehicle vibration signal was the vibration acceleration signal of the driver-seat system, set as ${\ddot{Z}}_{3}$). N is the dimension of the sample datum. The mathematical expressions of $f_{e1}$–$f_{e4}$ are presented below.

The mathematical expression of the average value of the absolute value is

(22) $$f_{e1}=\sum \left|{\ddot{Z}}_{3}\right|/N.$$

The mathematical expression of the standard deviation of the absolute value is

(23) $$f_{e2}=\sqrt{\sum {\left(\left|{\ddot{Z}}_{3}\right|-\sum \left|{\ddot{Z}}_{3}\right|/N\right)}^{2}/N}.$$

The mathematical expression of the maximum deviation of absolute value from the average value of the absolute value is

(24) $$f_{e3}=\max\left(\left|{\ddot{Z}}_{3}\right|-\sum \left|{\ddot{Z}}_{3}\right|/N\right).$$

The mathematical expression of the extreme difference of the absolute value is

(25) $$f_{e4}=\max\left(\left|{\ddot{Z}}_{3}\right|\right)-\min\left(\left|{\ddot{Z}}_{3}\right|\right).$$

### 2.5. ISA Algorithm and Comfortable Speed Strategy Formulation

Combined with Section 2.1 of this paper, the comfortable speed at five levels under different grade roads was calculated as the core step to form the comfortable speed strategy. As discussed in Section 2.1, the five levels of comfortable speed ($u_{a1}$–$u_{a5}$) correspond to the vehicle speed when the weighted root-mean-square acceleration value is 0.315, 0.5, 0.8, 1.25, and 2 m/s^2^, respectively. The conventional calculation involves a step-by-step solution, where the enumeration method is used to solve the corresponding speed of each grade road and each weighted root-mean-square acceleration value. This not only increases the workload of researchers, but also increases the calculation complexity to some extent. In addition, the differential equation of vehicle vibration with five degrees of freedom established by the Lagrange second equation is complex. This leads to difficulty when solving the speed.

In summary, this paper proposes a vehicle speed solution method based on the simulated annealing (SA) algorithm. In addition, this paper took A–E grade roads as examples to formulate the speed strategy. The SA algorithm was used to find the best speed value. The objective function of the optimization process was the relative error between the weighted root-mean-square acceleration value and its target value. In order to improve the convergence speed and precision of the algorithm, avoid the premature convergence of the algorithm, and improve the application effect of the algorithm in this case, this paper improved the standard SA algorithm (ISA algorithm) as follows:

(1) The SA algorithm was improved according to the verified steps of the engineering application effect in [39,40].
(2) The objective function was modified. Referring to the idea of parallel computing, the dimension of a single particle in the algorithm was set to 5, representing the comfortable speeds ($u_{a1}$–$u_{a5}$) for the same road grade. The objective function of the algorithm was modified to
  (26) $$F_{fitness1}=\sum_{i=1}^{5}\left|\frac{a_{wci}-a_{wi}}{a_{wi}}\right|\times 100\%,$$
  where $F_{fitness1}$ is the modified objective function, $a_{wci}$ is the measured value for the *i*-th weighted root-mean-square acceleration value, and $a_{wi}$ is the object value for the *i*-th weighted root-mean-square acceleration value.
(3) According to step (2), the improved algorithm could obtain five comfortable speeds of the same road grade in one operation. However, this would cause relatively high calculation complexity and increase the number of iterations, as each iteration of the algorithm would need to calculate the weighted root-mean-square acceleration value of the five speeds. Therefore, a switching variable was introduced into each sub-objective function of $F_{fitness1}$. The further improved objective function was as follows:
  (27) $$F_{fitness2}=\sum_{i=1}^{5}switch_{i}\left|\frac{a_{wci}-a_{wi}}{a_{wi}}\right|\times 100\%,$$
  where $switch_{i}$ is the switching variable of the *i*-th sub-objective function.

When a sub-objective function meets the accuracy requirement (i.e., a comfortable speed), the switching variable of the sub-objective function is 0 (thus closing the sub-objective function). The closed sub-objective function no longer participates in the subsequent iterative calculation of the algorithm.

(4) Prior information was introduced. The speed of the same vehicle is limited. Hence, when the same vehicle runs on the same grade road, its weighted root-mean-square acceleration value also has its own variation range. Therefore, this paper conducted simulation tests with the minimum speed and the maximum speed at the beginning of the algorithm execution to determine the upper and lower limits of the weighted root-mean-square acceleration value under the current road grade (the prior information of the algorithm). The number of sub-objective functions of the objective function was adjusted according to the prior information. For example, if the weighted root-mean-square acceleration value varied from 0.33 to 1.33, only $u_{a2}$–$u_{a4}$ (deleting sub-objective functions 1 and 5) at the current road grade were solved according to Section 2.1.

**Figure 7:**
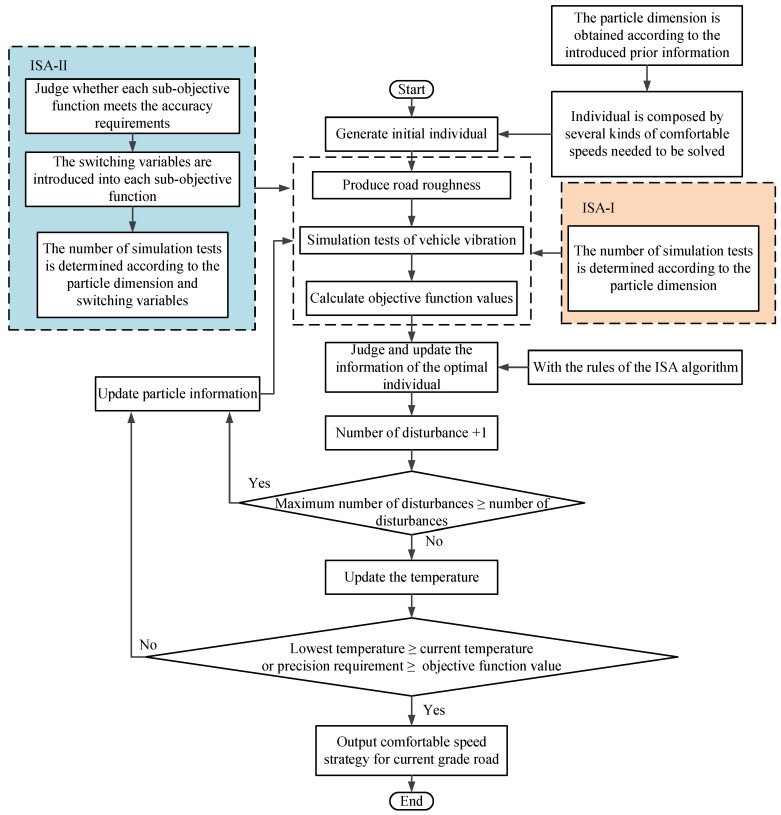
The flowchart of the two methods for obtaining the comfortable speed strategy.

In summary, according to the two different objective functions proposed in this paper, the ISA algorithm was denoted as the ISA-I algorithm and ISA-II algorithm. This study compared the application effects of the two improved algorithms. The flowchart of the two methods for obtaining the comfortable speed strategy is shown in Figure 7.

## 3. Results and Discussion

### 3.1. Simulation Results and Verification of Road Roughness Signal

**Figure 8:**
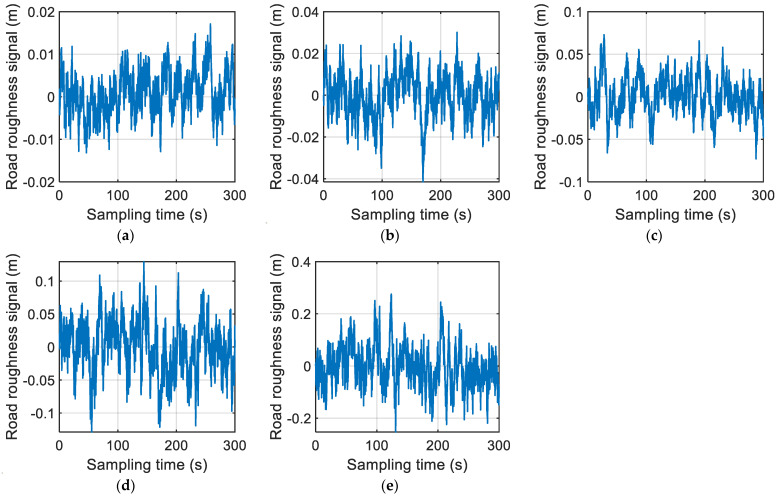
Simulated results of A–E grade road roughness signals: (**a**) class A; (**b**) class B; (**c**) class C; (**d**) class D; (**e**) class E.

In this paper, the A–E grade road roughness signals simulated using the filtering white noise method are shown in Figure 8 (the sampling time of each grade road roughness signal was 300 s, and the sampling frequency was 1024 Hz).

**Figure 9:**
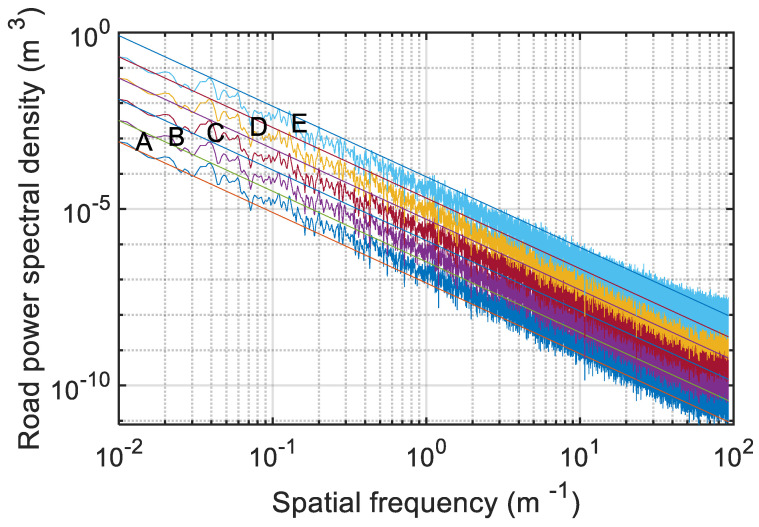
The verification results of A–E grade road roughness signals.

In this paper, the simulated road roughness signal in the time domain was converted to the road spatial power spectral density. According to the provisions of international standard ISO 8608, the verification results are shown in Figure 9.

The straight lines in Figure 9 are the upper and lower limits of the power spectral density values of each grade road. The maximum spatial frequency of road roughness is 5 or 10 m^−1^ [36]. The results show that, when the spatial frequency was 0.01–10 m^−1^, 90.88% of the A–E grade road simulation signals were within the standard limit. When the spatial frequency was 0.01–5 m^−1^, 91.53% of the A–E grade road simulation signals were within the standard limit. Therefore, the results show that the simulated roughness signals of each road grade were basically within the specified range. Therefore, the road roughness signal applied in this paper met the requirements.

### 3.2. The Road Recognition Results

**Figure 10:**
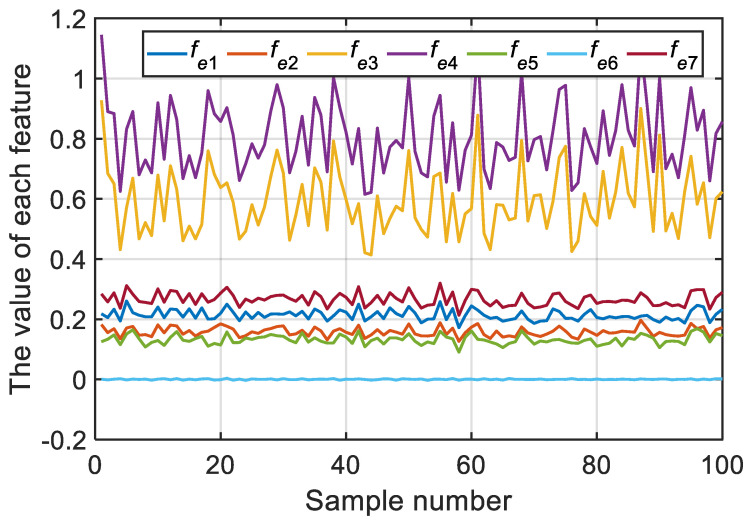
Seven features for 100 sample data under B grade road (as an example).

Taking B grade road as an example, the 100 sample data of vehicle vibration (a total of seven features) obtained in this study are shown in Figure 10.

According to Figure 10, the values and fluctuations of feature 6 (i.e., the average vibration acceleration signal of the driver-seat system) were too small. Therefore, feature 6 was deleted from the input features of the road recognition system.

The road recognition results using RF, ELM, and RBF-NN are shown in Figure 3 (processed using the same computer).

According to Table 3, RF and ELM had the highest recognition accuracy, followed by RBF-NN. RF took a long time, while ELM and RBF-NN took less time. The time consumption of this study refers to the sum of the training time and the time to get test results. ELM had a relatively optimal effect on recognition accuracy (both the training accuracy and the test accuracy were 100%) and execution speed (time consumption was reduced by 98.21% and 71.43%, respectively, compared with RF and RBF-NN). In summary, this research suggests using ELM for road recognition.

### 3.3. Results of Comfortable Speed Strategy Formulation

**Figure 11:**
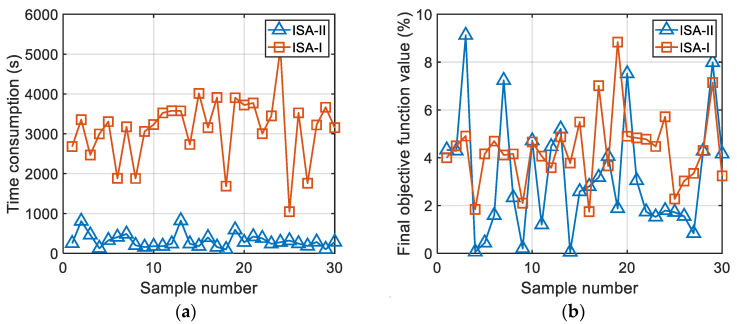
The comparative results of the two methods for obtaining comfortable speed strategy: (**a**) time consumption; (**b**) final objective function value.

The results comparison of the two methods for obtaining comfortable speed strategies (based on ISA-I algorithm and ISA-II algorithm, respectively) proposed in this paper is shown in Figure 11. The comparison of the two methods takes the comfortable speed strategy formulation of C grade road as an example. The application result of the heuristic intelligent optimization algorithm has a certain probability. Therefore, in this research, each method was run 30 times independently, and a comparative analysis was performed on the basis of the statistical results. This research set the iteration termination criterion of the algorithm as an objective function less than or equal to 5%.

According to Figure 11, it is obvious that the comfortable speed calculated using the two methods had high accuracy. The objective function results of the ISA-I algorithm and ISA-II algorithm were 3.20% and 4.34%, respectively. The objective function was the relative error according to Section 2.5. However, it is clear that the time consumption of ISA-II algorithm was relatively shorter. As the research was completed on the same computer, the ISA-II algorithm had a lower calculation complexity than ISA-I algorithm. Specifically, the average time consumption of the ISA-II algorithm and ISA-I algorithm was 310.46 s and 3119.23 s, respectively. The ISA-II algorithm reduced the time consumption by 90.05%.

**Figure 12:**
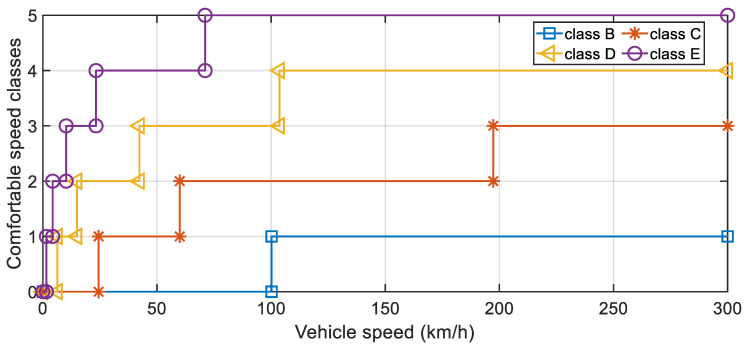
A–E grade road comfortable speed strategy for the research vehicle.

Therefore, this paper recommends the ISA-II algorithm to formulate the comfortable speed strategy. The results of the A–E grade road comfortable speed based on the ISA-II algorithm are shown in Figure 12 and Table 4. This research set the maximum speed of the vehicle to 300 km/h.

According to Table 4 and Figure 12, the speed corresponding to the same comfort level also decreased significantly with the decrease in road grade. Moreover, the variation relationship between the two was nonlinear. The research vehicle had good comfort performance on the A grade road, i.e., the passengers in the vehicle would not be uncomfortable due to the external road roughness excitation. When the research vehicle ran on B–E grade road, there would likely be discomfort among the passengers in the vehicle. In particular, for C–E grade road, serious discomfort could occur, affecting personal health (i.e., when the vehicle speed exceeded *u_a_*_3_).

**Figure 13:**
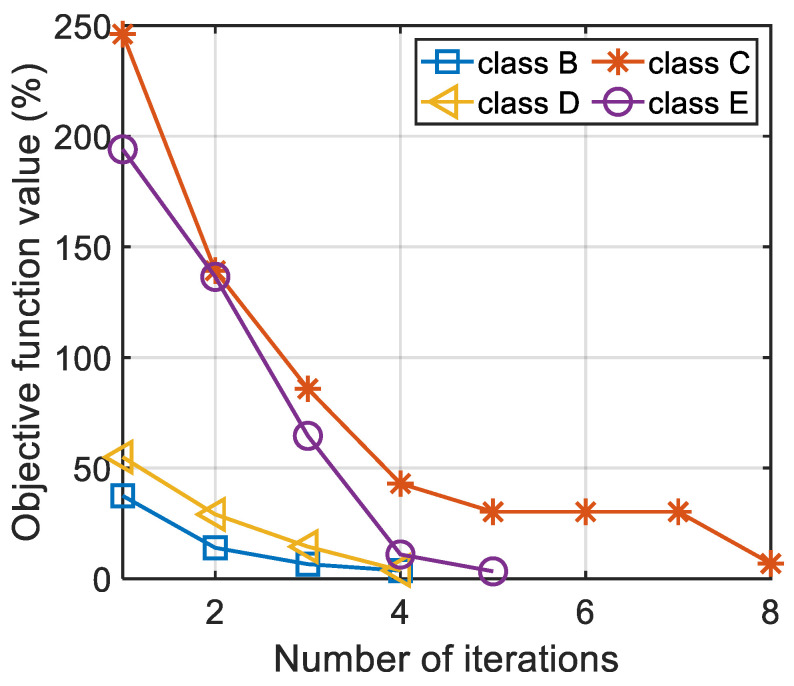
The algorithm iterative evolution curve for each grade road.

The algorithm iterative evolution curve for each grade road is shown in Figure 13.

**Figure 14:**
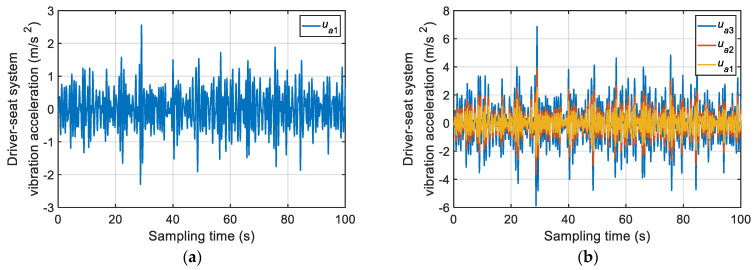

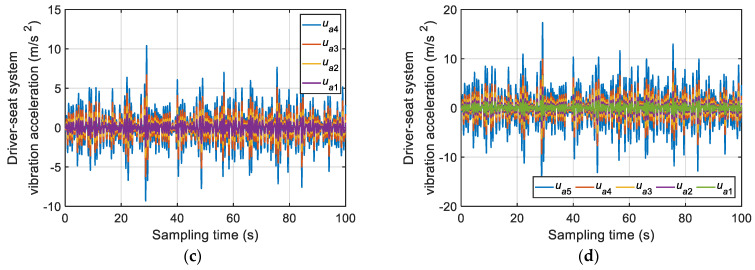
Time-domain signals of vertical vibration acceleration at driver-seat system under the comfortable speed: (**a**) class B; (**b**) class C; (**c**) class D; (**d**) class E.

According to Figure 13, the ISA-II algorithm proposed in this paper had a fast convergence speed. Only four, four, five, and eight iterations were needed to obtain the comfortable speed of B–E grade road, respectively. The final values (relative errors) of the objective function were 3.66%, 6.88%, 3.76%, and 3.33%, respectively. At the beginning of the algorithm iteration, the particles of the algorithm were generated randomly. Therefore, it can be found from Figure 13 that the initial relative error (the objective function value) was large. However, as the algorithm progressed, the evolution curve dropped rapidly. The final results show that only 5.25 iterations were needed to obtain the total comfortable speed of a vehicle on a certain grade road, and the relative error was 4.41%. In order to further verify the accuracy of the calculation results of comfortable speed, all comfortable speeds were substituted into the vehicle vibration model for the simulation test. The vertical vibration acceleration signal of the driver-seat system is shown in Figure 14. The results of the weighted root-mean-square acceleration value are shown in Table 5.

According to Figure 14 and Table 5, when the vehicle was driving at the obtained comfortable speed, the measured value of the weighted root-mean-square acceleration was basically consistent with the target value. The maximum relative error was 4.60%, the minimum relative error was 0.13%, and the average relative error was 2.06%. This verifies the correctness of the comfortable speed obtained using the ISA-II algorithm.

## 4. Conclusions

This paper proposed a comfortable speed strategy and studied its application technical route, aimed at providing speed control auxiliary decisions for drivers or autonomous driving systems from the perspective of passenger health and comfort. The research system was mainly composed of road recognition technology and a predeveloped comfortable speed strategy.

In terms of road recognition, by only measuring the vibration acceleration signal of the driver-seat system over 30 s, the road recognition method proposed in this paper could identify the road with high accuracy. Compared with RF and RBF-NN, ELM combined with six statistical characteristics of vehicle vibration signals had the highest road recognition accuracy and the shortest recognition time.

In terms of comfortable speed strategy formulation, this paper proposed two parallel computing methods based on the ISA algorithm. The ISA-II algorithm with the switching variable and prior information had a faster convergence speed while maintaining high accuracy. The ISA-II algorithm reduced the time consumption by 90.05%. Only 5.25 iterations were needed to obtain the total comfortable speed of a vehicle on a certain grade road, with a relative error of 4.41%. This study is helpful to the formulation of a comfortable speed strategy for a large number of different vehicles, and it can shorten the development cycle of vehicle speed control auxiliary decision systems.

## Figures and Tables

**Table 1:** The relationship between weighted root-mean-square acceleration value and people’s subjective feelings (referring to ISO 2631-1).

Weighted Root-Mean-Square Acceleration Value (m/s^2^)	People’s Subjective Feelings
<0.315	Comfortable
0.315~0.63	Some discomfort
0.5~1.0	Quite uncomfortable
0.8~1.6	Uncomfortable
1.25~2.5	Very uncomfortable
>2	Extremely uncomfortable

**Table 2:** The classification standard of eight grades of road roughness.

Road Grade	$G_{q}(n_{0})\cdot 10^{-6}(m^{3})$		
	Lower Limit	Geometric Mean	Upper Limit
A	8	16	32
B	32	64	128
C	128	256	512
D	512	1024	2048
E	2048	4096	8192
F	8192	16,384	32,768
G	32,768	65,536	131,072
H	131,072	262144	524,288

**Table 3:** Comparison of road recognition results of RF, ELM, and RBF-NN.

Method	RF	ELM	RBF-NN
Training accuracy	100%	100%	100%
Testing accuracy	100%	100%	99%
Time consumption (s)	1.12	0.02	0.07

**Table 4:** A–E grade road comfortable speed strategy for the research vehicle.

Comfortable Speed (km/h)	A	B	C	D	E
*u_a_*_1_	-	100.19	24.43	6.39	1.55
*u_a_*_2_	-	-	59.96	14.93	4.30
*u_a_*_3_	-	-	197.33	42.32	10.22
*u_a_*_4_	-	-	-	103.70	23.29
*u_a_*_5_	-	-	-	-	71.07

**Table 5:** The validation results of the weighted root-mean-square acceleration value.

Test Number	Road Grade	Driving Speed (km/h)	Measured Value of *a_w_* (m/s^2^)	Target Value of *a_w_* (m/s^2^)	Relative Error (%)
1	B	100.19	0.306	0.315	2.86
2	C	24.43	0.311	0.315	1.27
3	C	59.96	0.482	0.500	3.60
4	C	197.33	0.801	0.800	0.13
5	D	6.39	0.318	0.315	0.95
6	D	14.93	0.486	0.500	2.80
7	D	42.32	0.815	0.800	1.88
8	D	103.70	1.244	1.250	0.48
9	E	1.55	0.314	0.315	0.32
10	E	4.30	0.522	0.500	4.40
11	E	10.22	0.805	0.800	0.63
12	E	23.29	1.214	1.250	2.88
13	E	71.07	2.092	2.000	4.60

## Data Availability

The data presented in this study are available on demand from the corresponding author or first author (900231@nuist.edu.cn or cz38@njfu.edu.cn).
